# Microstructural and Surface Texture Evaluation of Orthodontic Microimplants Covered with Bioactive Layers Enriched with Silver Nanoparticles

**DOI:** 10.3390/jfb15120371

**Published:** 2024-12-09

**Authors:** Magdalena Sycińska-Dziarnowska, Magdalena Ziąbka, Katarzyna Cholewa-Kowalska, Gianrico Spagnuolo, Hyo-Sang Park, Steven J. Lindauer, Krzysztof Woźniak

**Affiliations:** 1 Department of Maxillofacial Orthopaedics and Orthodontics, Pomeranian Medical University in Szczecin, Al. Powst. Wlkp. 72, 70111 Szczecin, Poland; 2 Department of Ceramics and Refractories, Faculty of Materials Science and Ceramics, AGH University of Krakow, al. A. Mickiewicza 30, 30059 Krakow, Poland; 3 Department of Glass Technology and Amorphous Coatings, Faculty of Materials Science and Ceramics, AGH University of Krakow, 30059 Krakow, Poland; 4 Department of Neurosciences, Reproductive and Odontostomatological Sciences, University of Naples “Federico II”, 80131 Napoli, Italy; 5 School of Dentistry, College of Dental Medicine, Kaohsiung Medical University, Kaohsiung 80708, Taiwan; 6 Department of Orthodontics, College of Dentistry, Kyungpook National University, Daegu 41940, Republic of Korea; 7 Department of Orthodontics, School of Dentistry, Virginia Commonwealth University, Richmond, VA 23103, USA

**Keywords:** microstructural evaluation, surface modification, orthodontic microimplants, silver nanoparticles

## Abstract

Bacterial infections are a common cause of clinical complications associated with the use of orthodontic microimplants. Biofilm formation on their surfaces and subsequent infection of peri-implant tissues can result in either exfoliation or surgical removal of these medical devices. In order to improve the properties of microimplants, hybrid coatings enriched with silver nanoparticles, calcium, and phosphorus were investigated. The present study aimed to assess the microstructure of commercially available microimplants composed of a medical TiAlV (Ti6Al4V) alloy covered with organic–inorganic layers obtained by the sol–gel method using the dip-coating technique. The microstructures and elemental surface compositions of the sterile, etched, and layer-modified microimplants were characterized by scanning electron microscopy with X-ray energy-dispersive spectroscopy (SEM-EDS). Elements such as silver (Ag), calcium (Ca), phosphorus (P), silicon (Si), oxygen (O), and carbon (C) were detected on the microimplant’s surface layer. The SEM observations revealed that control microimplants (unetched) had smooth surfaces with only manufacturing-related embossing, while etching in hydrofluoric acid increased the surface roughness and introduced fluoride onto the microimplants. Layers with only silver nanoparticles reduced the roughness of the implant surface, and no extrusion was observed, while increased roughness and emerging porosity were observed when the layers were enriched with calcium and phosphorus. The highest roughness was observed in the microimplants etched with AgNPs and CaP, while the AgNPs-only layer showed a reduction in the roughness average parameter due to lower porosity. Enhancing the effectiveness of microimplants can be achieved by applying selective surface treatments to different parts. By keeping the outer tissue contact area smooth while making the bone contact area rough to promote stronger integration with bone tissue, the overall performance of the implants can be significantly improved.

## 1. Introduction

Orthodontic microimplants are becoming increasingly popular as a treatment option to strengthen anchorage during orthodontic treatment and to facilitate individually planned movement of specific groups of teeth [1]. Despite their benefits, microbial control is critical to their success as bacterial infection can lead to microimplant failure [2].

Titanium and its alloys, particularly Ti6Al4V (titanium–aluminum–vanadium), are widely used in biomedical implants due to their excellent mechanical properties, biocompatibility, and corrosion resistance [3,4]. Temporary anchorage devices (TADs) composed of Ti6Al4V play an important role in orthodontic treatment, providing stable support for tooth movement and alignment [3,5]. To further improve their functionality and biological integration, surface modifications can be applied. Changes in surface roughness, chemical composition, and the incorporation of bioactive elements such as silver (Ag), calcium (Ca), and phosphorus (P) can significantly improve the partial osseointegration process, resulting in greater implant stability and enhanced antimicrobial properties [6].

Among the surface modification techniques, the sol–gel method has gained particular attention due to its ability to produce hybrid organic–inorganic coatings with customizable properties [7]. These coatings can be designed to incorporate nanoparticles, such as silver nanoparticles (AgNPs), which have been shown to have strong antibacterial activity [8].

The surface modification of biomedical materials is crucial for improving both osseointegration and resistance to bacterial infection [9]. Techniques incorporating bioactive additives such as silver, gold, copper, and chitosan biopolymers are key to enhancing antimicrobial resistance [10,11,12,13,14,15]. Studies on microimplants and implants have demonstrated the successful integration of AgNPs into surface coatings [16,17,18,19,20,21]. In addition, the incorporation of calcium and phosphorus into these coatings can promote partial osseointegration, thereby supporting bone formation around the implant [17,22].

The sol–gel process combined with dip-coating enables precise control over the application of these coatings, ensuring a uniform and adherent layer on the microimplant surface. Partial osseointegration is a distinct advantage in orthodontic applications since it provides a solution that combines stable anchorage with ease of insertion and removal [23]. Ultimately, the goal of these modifications is to optimize the interaction between the microimplant surface and bone, reduce bacterial colonization, and minimize the risk of biofilm formation while promoting long-term microimplant stability.

The present study aims to evaluate the microstructural characteristics of commercially available Ti6Al4V microimplants that have undergone surface modification using the sol–gel method. The modifications include etching with hydrofluoric acid (HF) to increase surface roughness and coating with hybrid layers containing AgNPs, with or without calcium and phosphorus components. The surface microstructures and elemental compositions of both unmodified and modified microimplants are characterized using scanning electron microscopy (SEM) coupled with energy-dispersive spectroscopy (EDS), and confocal laser scanning microscopy. Understanding the effects of these modifications is critical to optimizing microimplant surfaces for improved clinical outcomes, such as increased stability and resistance to microbial colonization. The study presents a novel approach by incorporating silver nanoparticles. The innovation involves the post-firing stage, where the organic matrix on the microimplant surface generates a distinctive layer containing silver nanoparticles, as well as calcium and phosphorus, thus inducing the formation of an apatite layer on the implant’s surface. This novel approach aims to improve the antimicrobial properties and create a favorable environment for better stability.

The objective of this study is to evaluate and compare the effects of different surface treatment methods on the roughness, microstructures, and chemical compositions of orthodontic microimplant surfaces. This investigation aims to determine the effects of different treatment methods, including etching, sol–gel coating with AgNPs, and hybrid layers enriched with Ca and P, on the overall surface characteristics of the microimplants.

## 2. Materials and Methods

### 2.1. Hybrid Layer Manufacturing on Microimplants

Microimplants (Orthodontic Temporary Anchorage Device system self-drilling thread with a diameter of 1.4 mm) composed of the Ti6Al4V alloy were manufactured by Dentos (Dalseo-Gu, Daegu, Republic of Korea). The microimplants investigated in this study, measuring 10 mm × 1.4 mm, are smaller than conventional dental implants and have a rougher surface texture. The sample of 12 microimplants was divided into different groups, as shown in Table 1. Control and surface analysis as well as reproducibility characterization were performed on 6 samples each (1 per group).

Hybrid (organic–inorganic silicate) sols for surface coating were prepared by the sol–gel method. The chemical composition of layers was designed with 0.5 mol % concentrations of metallic precursors. The coating solution was prepared using the following precursors: TEOS (tetrethylorthosilicate, Si(OC_2_H_5_)_4_); TMSPM (3-(Trimethoxysilyl) propyl methacrylate, H_2_C=C(CH_3_)CO_2_(CH_2_)_3_Si(OCH_3_)_3_); TIP (titanium (IV) isopropoxideTi(OC_3_H_7_)_4_); and nitrates AgNO_3_ as a source of metallic ions. First, the metallic precursor was dissolved in propanol, serving as a solvent. The solution was mixed for 30 min in a magnetic stirrer. Next, TEOS, TMSPM, and TIP were successively added to the solution. Having introduced each of the reagents, we then stirred the solution for 30 min. The aqueous solution of the HNO_3_ nitric acid was used as a reaction catalyst. The volume ratio of all the components to the propanol solvent was 1:8. A solution containing calcium and phosphorus precursors was prepared using triethylphosphate (TEP; OP(OC_2_H_5_)_3_) and calcium nitrate tetra-hydrate (Ca(NO_3_)_2_*4H_2_O) as a source of P_2_O_5_ and CaO by mixing appropriate amounts of the components (for obtaining a Ca/P molar ratio in solution close to 1.67) in propanol, and then the hybrid sol was added in volume ratio 1:5. Prior to layering, the solution aging time was 24 h, and the viscosity equaled 6 cP. The layers were applied by the dip-coating technique with the 20 mm/min withdrawal speed on the surface of base and etched in HF microimplants. Acid etching was intended to increase the surface roughness of microimplants for better adhesion of layers. Then, the coated microimplants were dried in ambient conditions for 24 h. Next, the applied layers were stabilized by the two-step thermal treatment at 80 °C for 10 min and 130 °C for 15 min (layers enriched with silver) and thermal treatment at 80 °C for 10 min; 130 °C for 15 min and 500 °C for 20 min (layers enriched with calcium, phosphorus, and silver). The following nomenclature was adopted to standardize the names of the samples in this work (Table 1).

### 2.2. Material Examinations

The primary outcome of the study was to evaluate the surface roughness and microstructures of the treated microimplants, as well as their elemental compositions after surface treatment.

The surface roughness of the microimplants was evaluated by SEM and confocal microscopy. A comparison of treated and untreated surfaces was developed to identify morphological changes (cracks, porosity, and embossing). Analysis of the presence and weight percentage of key elements (e.g., silver, calcium, and phosphorus) was performed using EDS.

#### 2.2.1. Scanning Electron Microscopy Coupled with Energy-Dispersive Spectroscopy

SEM and confocal microscopy enabled high-resolution imaging of the microstructures and surface topography of the microimplants. This is critical for observing the effects of etching and coating on surface roughness and the presence of porosity and cracks in the bioactive layers, as well as the overall adhesion of the layer to the implant surface.

EDS provides qualitative and semi-quantitative analysis of the elemental composition of bioactive layers. It has been used to confirm the incorporation of silver, calcium, and phosphorus nanoparticles regarding the coatings. The accelerating voltage of the electron beam during EDS analysis was selected to excite all the elements present in the layers.

The detailed microstructure examination of the uncovered, unetched, and coated microimplants with hybrid layers was performed using a scanning electron microscope SEM—Apreo 2S Low vac, Thermo Fisher Scientific (Waltham, MA, USA) with APEX Advanced Software (26 May 2022, No 2.5.1001.0001), for EDS, EDAX. The observations were carried out in low- and high-vacuum conditions using LVD and ETD detectors and 10 or 15 kV accelerating voltage for observation and EDS analysis, respectively. 

#### 2.2.2. Confocal Microscopy

Confocal microscopy enabled precise evaluation of the roughness average (Ra) parameter, which is a critical factor in assessing the suitability of microimplants for partial osseointegration and bioactivity. Based on previous research, an optimal Ra for this study was determined to range between 0.2 µm and 1.0 µm. This range supports cellular adhesion while minimizing excessive bacterial colonization. Surface roughness values below 0.2 µm are considered to be inadequate as they can lead to reduced surface interactions, compromising material integration and bioactivity. Studies suggest no additional benefit for surfaces smoother than 0.2 µm [20]. Conversely, Ra values greater than 1.0 µm may increase the risk of bacterial colonization and lead to complications in clinical applications. While some studies have reported Ra values above 2.0 µm as inappropriate for medical applications, for the purposes of this research, more stringent criteria were adopted, limiting the Ra threshold to 1.0 µm to account for gingival and subgingival applications and to ensure that the observed Ra values are consistent with their biological and clinical implications.

The surface topography of microimplants was observed using a laser confocal microscope Lext OLS 4000 (Olympus, Tokyo, Japan) with the magnification 50×. The scanned area for each measurement was 640 × 640 µm, providing a detailed representation of the microimplant surface. The confocal microscopy technique provides a noncontact approach to measuring surface features, ensuring that the coatings on the microimplants remain undisturbed during analysis.

## 3. Results

**Figure 1:**
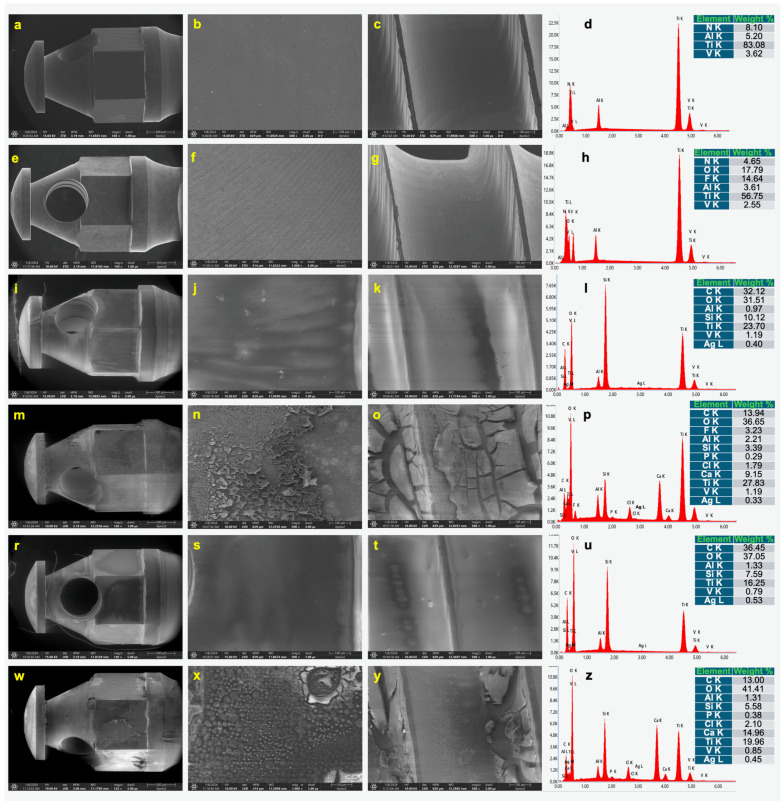
SEM images of microimplant surface and EDS analysis of Ti (**a**–**d**), Ti-etched (**e**–**h**), Ti- etched with layer with AgNPs (**i**–**l**), Ti-etched with layer with AgNPs and enriched with CaP (**m**–**p**), Ti with layer with AgNPs (**r**–**u**), and Ti with layer with AgNPs and enriched with CaP (**w**–**z**). Magnification: 130×, 500×, and 1000×.

**Figure 2:**
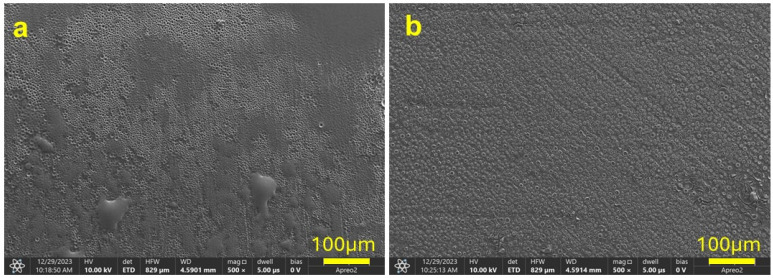
SEM images of plate samples composed of Ti6Al4V alloy: layer with AgNPs (**a**) and layer with AgNPs and CaP (**b**). Magnification: 500×.

Based on the SEM observations of the microimplants, for the control microimplants, it was noted that both the screw head and thread surfaces were smooth, displaying only embossing due to the manufacturing process (Figure 1a–d), clearly visible at higher magnifications. This serves as a baseline for comparison. The layers were also applied to the same alloy but to the surfaces of flat plates (Figure 2). The EDS analysis confirmed the chemical composition of the Ti6Al4V alloy, which was most likely nitrided.

**Figure 3:**
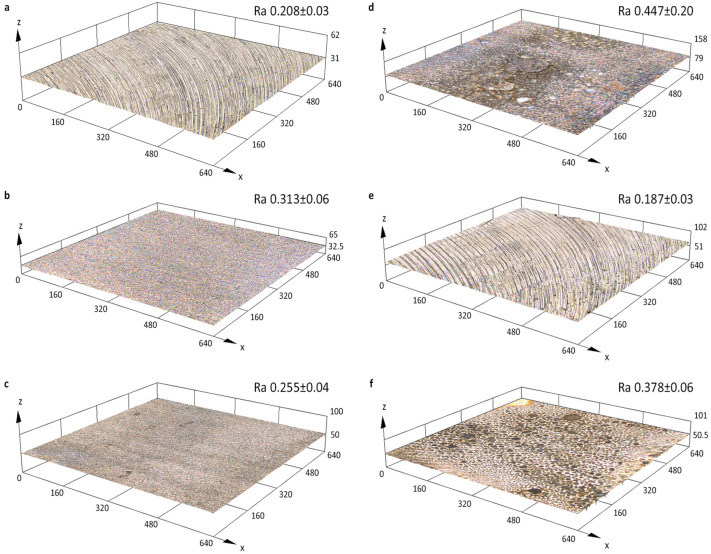
Confocal laser scanning microscopy images of microimplant surfaces showing the roughness (Ra) of Ti (**a**), Ti-etched (**b**), Ti-etched with layer with AgNPs (**c**), Ti-etched with layer with AgNPs and enriched with CaP (**d**), Ti with layer with AgNPs (**e**), and Ti with layer with AgNPs and enriched with CaP (**f**).

Etching the microimplants in hydrofluoric acid affected their roughness (Figure 1e–g and Figure 3b). The SEM images demonstrated more visible post-etching embossing. In the table and on the EDS spectrum, fluoride is also present on the surface of the microimplant (Figure 1h). Coating the microimplants with a layer containing AgNPs had a major effect on the surface topography, with no embossing being observed, and the layer was partially amorphous (Figure 1i–k). The coatings with AgNPs appeared to be smoother, indicating a good distribution of the bioactive layer.

On the other hand, the EDS analysis confirmed the presence of elements such as carbon, oxygen, silicon, and silver in the assumed weight percentage (Figure 1l). The layer containing AgNPs and enriched with phosphorus and calcium due to the higher temperature of the final thermal treatment process (500 °C) was characterized by the occurrence of high porosity and cracks (Figure 1m–o), which essentially translates into higher roughness (Figure 3d).

A phenomenon of this nature and magnitude was not observed when the layers were applied to the same alloy but to the surfaces of flat plates (Figure 2). This demonstrates the limitation of the final heat treatment process on the quality and adhesion of the layers to threads. The EDS analysis confirmed the presence of calcium and phosphorus; however, it was observed that phosphorus was not stable during the heat treatment process, and its content was less than 0.5% by weight (Figure 1p). The microstructure of the surface of the microimplants not etched with a silver layer (Figure 1r–u) shows a similar topographic character to that of the microimplants etched with a silver layer. In contrast, the surface of the microimplants not etched with a layer of silver and enriched with CaP (Figure 1w–z) demonstrated a similar topographic character to the surface of those microimplants with the same layer but not etched, with the difference that the layer was less cracked and adhered better to the surfaces of both the microimplant head and thread.

Figure 2 shows the surfaces of plates coated with layers containing AgNPs (Figure 2a) and AgNPs and CaP (Figure 2b). The coatings on the flat plates provided additional context for interpreting the behavior of the coatings on curved microimplant surfaces. The images show that the surfaces of both plates were completely covered, confirming proper layer formation. No cracks are visible on either plate, only porosity, which was higher for the CaP-rich layer. The higher porosity observed for the layer with AgNPs and CaP is consistent with the thermal treatment effects observed in the microimplants.

The confocal microscopy results regarding the surface roughness of the microimplants are in agreement with the SEM observations. An increase in roughness was observed by both etching (Figure 3b) and coating (Figure 3c,d,f). The microimplant etched with a layer containing AgNPs and CaP (Figure 3d) showed the highest roughness compared to the control microimplant (Figure 3a), whereas the layer containing only AgNPs showed a reduction in the roughness average (Ra) parameter due to lower porosity (Figure 3e).

The Ra values for the microimplants were measured as follows (Figure 3): Ti (a): Ra = 0.208 ± 0.03 µm, Ti-etched (b): Ra = 0.313 ± 0.06 µm, Ti-etched with a layer containing AgNPs (c): Ra = 0.255 ± 0.04 µm, Ti-etched with a layer containing AgNPs and enriched with CaP (d): Ra = 0.447 ± 0.20 µm, Ti with a layer containing AgNPs (e): Ra = 0.187 ± 0.03 µm, and Ti with a layer containing AgNPs and enriched with CaP (f): Ra = 0.378 ± 0.06 µm.

Five of the six samples fell within the optimal Ra range established for this study, which is expected to support reduced bacterial colonization and improve biological performance. The non-etched sample with an AgNPs coating (e) exhibited an Ra value of 0.187 ± 0.03 µm, which is slightly below the optimal range but remains close to the expected values for favorable surface interaction.

The Ra parameters suggest that different surface modifications result in distinct changes in roughness, which, in turn, affect the potential for partial osseointegration, mechanical stability, and bioactivity. The HF acid etching produced a rough surface with optimal Ra values for improving the mechanical interlocking and osseointegration. However, the coating with AgNPs reduced the roughness, which may be beneficial regarding the antimicrobial properties but less favorable in terms of the initial bone–implant contact.

## 4. Discussion

The present study investigated the microstructural properties of Ti6Al4V microimplants subjected to various surface modifications, including acid etching and hybrid organic–inorganic coatings containing AgNPs, with or without calcium (Ca) and phosphorus (P). The effects of these modifications were evaluated using scanning electron microscopy (SEM), energy-dispersive spectroscopy (EDS), and confocal laser scanning microscopy to understand their impact on the surface roughness, chemical composition, and potential clinical applications.

Between 2001 and 2020, a substantial number of 2346 publications on peri-implantitis were documented in the Web of Science Core Collection, underscoring the importance and complexity of this topic, with a primary focus on epidemiology and therapeutic interventions to combat peri-implantitis [24]. To the best of our knowledge, only three in vitro studies have addressed the issue of silver nanoparticle coatings in orthodontic microimplants [16,17,18]. In the study by Subramanian et al., Ti-BP-AgNPs demonstrated effective antibacterial properties against *Lactobacillus* and *S. aureus*, while discernible antibacterial activity against *S. mutans* was observed. In contrast, a study by Venugopal et al. reported no antimicrobial effect for Ti-AgNPs-enriched microimplants. However, the authors did not report the statistical significance of their results and did not investigate the effect of roughness on the microbial results. Therefore, we started our research with an analysis of the surface structure and roughness to enable a focused study regarding the antimicrobial effects in the next phase of our investigation.

### 4.1. Effect of Acid Etching on Microstructure and Roughness

HF acid etching resulted in a significant increase in surface roughness, with Ra values of 0.313 ± 0.06 µm measured for the etched microimplants (Figure 3b). This increase in roughness was observed in both SEM and confocal microscopy images, where the underlying microstructure of the Ti6Al4V alloy was exposed, resulting in a more pronounced and rougher surface compared to the unetched microimplants (Ra = 0.208 ± 0.03 µm). This roughened surface is particularly beneficial for enhancing partial osseointegration as increased surface roughness promotes better mechanical interlock between the microimplant and surrounding bone tissue, thus potentially improving the microimplant stability [6,25]. The presence of residual fluoride on the etched surfaces, as detected by the EDS analysis, may require careful consideration due to its potential impact on long-term biocompatibility [26].

### 4.2. Effects of AgNP-Containing Coatings on Surface Topography

The application of a hybrid layer containing AgNPs resulted in a significant reduction in the surface roughness of unmodified commercial implants, with Ra values of 0.255 ± 0.04 µm (Figure 3c). The reduction in roughness can be attributed to the partially amorphous nature of the applied hybrid layer, which tends to fill in surface irregularities. The EDS analysis confirmed the successful incorporation of AgNPs, along with carbon, oxygen, and silicon, indicating the formation of a complex hybrid matrix. The presence of silver provides a potential antimicrobial benefit, reducing the risk of peri-implant infection [13]. However, the smoother surface, despite its potential antimicrobial properties, may limit mechanical interlocking and could be less favorable for initial bone–implant contact than the rougher etched surfaces.

### 4.3. Impact of CaP-Enriched Layers on Microstructure

The addition of calcium and phosphorus to the AgNPs-containing hybrid layers resulted in significant changes to the microstructure and surface roughness. The SEM analysis revealed a more porous and cracked surface morphology after the final thermal treatment at 500 °C (Figure 1m–o), which was reflected in the Ra value that increased to 0.447 ± 0.20 µm (Figure 3d). This high porosity could potentially enhance bone cell attachment and growth, making these coatings beneficial for partial osseointegration. However, the increased roughness and presence of cracks could also pose challenges for mechanical stability as the cracked regions could act as stress concentrators under loading conditions, potentially affecting the longevity of the coating [27].

The EDS analysis showed that, while calcium was retained in the coating, the phosphorus content decreased significantly after the thermal treatment. This suggests that phosphorus may not be stable at higher temperatures, resulting in its partial evaporation or decomposition during the heating process. This instability of phosphorus could affect the bioactive potential of the coating as the Ca/P ratio is critical for promoting bone growth [28]. Future studies may need to optimize the thermal treatment process to better retain phosphorus and improve the bioactivity of the coating.

### 4.4. Comparison Between Etched and Non-Etched Coatings

A comparison between etched and non-etched microimplants coated with the same hybrid layers demonstrated differences in surface adhesion and crack formation. The coatings applied to etched surfaces displayed a higher degree of cracking, likely due to the increased surface roughness and the stress introduced during thermal expansion and contraction. In contrast, the coatings on non-etched surfaces showed fewer cracks and better adhesion (Figure 1w–z). This suggests that, while etching may improve the mechanical interlock of the base material, it may create a more challenging substrate for the application of certain coatings, particularly those requiring high-temperature treatment.

### 4.5. Clinical Implications and Future Directions

The findings of this study underscore the complexity of optimizing microimplant surfaces for clinical use. Each modification, etching, AgNPs incorporation, and CaP enrichment presents distinct advantages and challenges. For example, the antimicrobial properties of silver can be beneficial in reducing the risk of infection [8], but the smoother surface resulting from AgNPs-containing layers may limit the potential for partial osseointegration. On the other hand, CaP-enriched coatings can improve bioactivity and integration with bone tissue but may suffer from stability issues due to the cracks formed during the thermal treatment.

These results suggest that a tailored approach to surface modification, taking into account the specific clinical application and the required balance between antimicrobial activity and partial osseointegration, is essential. Future research could focus on refining the sol–gel process to improve the uniformity and stability of coatings, as well as exploring alternative methods for phosphorus stabilization at higher temperatures.

Antimicrobial studies are needed to fully evaluate the effectiveness of surface modifications and coatings on microimplants. These evaluations will provide insight into how the enhanced surface properties affect microbial behavior and infection prevention. The antibacterial potential of the silver coatings is an important feature, providing a robust defense against a wide range of bacterial pathogens, including antibiotic-resistant strains. The silver ions released from the coating interact with bacterial cell walls, disrupting cell function and causing cell death. This mechanism not only effectively reduces bacterial colonization on the coated surface but also contributes to long-lasting antimicrobial properties, making it particularly valuable in applications where hygiene and sterility are critical [29,30]. In addition, in vivo studies would be valuable to validate the long-term performance of these modified microimplants and to assess their behavior under physiological conditions.

The study highlights several strengths, such as the introduction of an innovative coating, and its alignment with the growing need for controlled partial osseointegration in microimplants.

## 5. Conclusions

Overall, this study highlights the significant impact of surface treatments on the microstructural properties of Ti6Al4V microimplants. The conclusions drawn from the observations indicate that the coatings produced have the potential for improved performance. However, the application process using the dip-coating technique needs to be modified to increase the AgNPs content and produce thinner layers that adhere more effectively to the microimplant surface. The incorporation of calcium and phosphorus into the layer may offer the potential for establishing a better connection at the microimplant–bone boundary. However, it also increases the roughness and porosity of the surface. To address this challenge, selective surface treatment of different parts of the microimplants may enhance their effectiveness by providing a smooth bactericidal surface on the outer tissue contact area while creating a rougher surface on the bone contact area to promote stabilization. The potential of bioactive layers enriched with silver nanoparticles used on microimplants promises to improve patient outcomes, increase the safety of orthodontic treatment, and ensure the long-term success of microimplant interventions.

## Figures and Tables

**Table 1:** Sample nomenclature.

Microimplant Characteristics	Microimplant Nomenclature
Titanium alloy Ti-6Al-4V unetched	Ti
Titanium alloy Ti-6Al-4V etched in HF	Ti-E
Titanium alloy Ti-6Al-4V etched in HF and covered with hybrid layer containing 0.5 mol % AgNPs	Ti-E-Ag
Titanium alloy Ti-6Al-4V etched in HF and covered with hybrid layer containing 0.5 mol % AgNPs and containing Ca and P	Ti-E-Ag-CaP
Titanium alloy Ti-6Al-4V unetched and covered with hybrid layer containing 0.5 mol % AgNPs	Ti-Ag
Titanium alloy Ti-6Al-4V unetched and covered with hybrid layer containing 0.5 mol % AgNPs and containing Ca and P	Ti-Ag-CaP

## Data Availability

The original contributions presented in the study are included in the article; further inquiries can be directed to the corresponding author.
